# Community-driven type 2 diabetes prevention in primary healthcare: a mixed-methods pre-post intervention study in Thailand

**DOI:** 10.1186/s12875-026-03362-x

**Published:** 2026-05-12

**Authors:** Benjayamas Pilayon, Arunrat Utaisang, Wilawan Bunkon, Phatcharaporn Kawansu, Pipatpong Kempanya, Kanin Chueaduangpui, Pongtorn Wongsawan, Nitikorn Phoosuwan

**Affiliations:** 1 Department of Community Health Nursing, Boromarajonani College of Nursing Nakhon Phanom, Nakhon Phanom University, Nakhon Phanom, 48000 Thailand; 2 Department of Adult and Aging Nursing, Boromarajonani College of Nursing Nakhon Phanom, Nakhon Phanom University, Nakhon Phanom, 48000 Thailand; 3 Nakhon Phanom Hospital, Nakhon Phanom, 48000 Thailand; 4 Phonsawan Hospital, Nakhon Phanom, 48190 Thailand; 5 Faculty of Liberal Arts and Science, Nakhon Phanom University, Nakhon Phanom, 48000 Thailand; 6 Faculty of Public Health, Thammasat University, Khlong Luang , Pathum Thani 12121 Thailand; 7 Department of Public Health and Caring Sciences, Uppsala University, Box 564, Uppsala, SE-75122 Sweden

**Keywords:** Community-based diabetes prevention, Community health workers, Health system strengthening, Health system transformation, Primary healthcare

## Abstract

**Background:**

The prevalence of type 2 diabetes (T2D) in low- and middle-income countries demands innovative primary healthcare approaches to disease prevention. This study examined a community-driven type 2 diabetes prevention model (CDDP-model) through primary healthcare settings in a northeast province in Thailand, and analyzed using the World Health Organization Framework: Six building blocks (SBBs).

**Methods:**

A mixed-methods single-arm pre-post intervention study with convergent design was conducted in two sub-districts representing rural and peri-urban contexts. There were 80 people at risk of T2D participated in the CDDP-model for eight weeks and followed-up in the 12th and 24th Weeks. Quantitative data included anthropometric measurements, clinical outcomes, and knowledge assessments, where qualitative data comprised focus group discussions and interviews. Data were analyzed using analysis of variance (ANOVA) with 95% significant level. Thematic analysis was applied.

**Results:**

The model demonstrated improvements in health outcomes and perceived enhancements. Governance shifted from top-down management to participatory community co-design. SBBs identified: (1) Health information systems evolved; (2) Local resource mobilization expanded; (3) Service delivery networks is integrated; (4) Health workforce transformation included; and (5) Technology adoption encompassed. Diabetes prevention knowledge scores increased with clinical outcomes. Qualitative findings revealed (1) Enhanced community ownership, (2) Peer support networks, (3) Sustained behavioral change mechanisms, and (4) Strengthened community-primary care relationships.

**Conclusions:**

This community-driven diabetes prevention model is feasible and acceptable in primary healthcare settings, with promising short-term improvements in selected health outcomes. However, further controlled and long-term studies are needed to confirm effectiveness and system-level impact.

**Supplementary Information:**

The online version contains supplementary material available at 10.1186/s12875-026-03362-x.

## Introduction

Type 2 Diabetes (T2D) represents one of the most pressing global health challenges of the 21st century, with prevalence rates escalating particularly in low- and middle-income countries (LMICs) undergoing rapid urbanization and lifestyle transitions [1]. In Thailand, T2D burden has reached epidemic proportions, affecting approximately 8–10% of the adult population, with significantly higher rates in urban and peri-urban communities [2]. Traditional healthcare approaches, predominantly focused on clinical management in tertiary facilities, have proven insufficient to address the complex socio-ecological determinants of diabetes in primary care and community settings [3].

Recent paradigm shifts in global health emphasize the critical role of primary healthcare systems and community engagement in preventing and managing chronic diseases [4, 5]. World Health Organization (WHO) identifies six essential building blocks (SBBs)—service delivery, health workforce, information systems, medical products and technologies, financing, and leadership/governance—as foundational elements requiring systematic strengthening in primary care contexts [6]. However, operationalizing this framework within community-based primary care necessitates transformative change processes that fundamentally alter power relationships, knowledge systems, and resource allocation mechanisms.

### Theoretical framework: Transformation change in primary healthcare

Transformation change in healthcare system requires fundamental improvements in structures, relationships, and mindsets rather than incremental modifications to existing practices [7, 8]. Unlike reform-oriented approaches that maintain existing power hierarchies, transformation change in primary healthcare emphasizes: (1) redistribution of decision-making from centralized institutions to community stakeholders and primary care providers, (2) integration of professional and experiential knowledge systems, (3) reconfiguration of resource flows to support community-generated priorities within primary care, and (4) development of new capabilities enabling communities to function as co-producers of health alongside primary care teams [9, 10]. Therefore, this framework is intended to analyze the effects of a community-based diabetes prevention program integrated within primary healthcare and how to strengthen healthcare system at the primary care level. The outcomes of the study included (1) clinical outcomes of the people at risk of T2D, (2) behavioral outcomes of the people at risk of T2D, and (3) primary healthcare system transformation.

### Research objectives

This mixed-method study aimed to: (1) implement and evaluate a five-dimensional community-driven type 2 diabetes prevention program integrated within primary healthcare settings, (2) analyze transformation change processes across WHO SBBs in primary care contexts, (3) document mechanisms enabling community ownership and sustained behavioral change within primary healthcare systems, and (4) generate evidence-based recommendations for scaling community-based diabetes prevention within primary healthcare systems in similar LMIC contexts. The mixed-method study covered: a quantitative design confirmed by statistics and mass of people, whilst a descriptive qualitative design was used to explore and describe the direct feelings and experiences of participants [11].

## Methods

### Study context and primary healthcare setting

Nakhon Phanom province, located in northeastern Thailand along the Mekong River, exemplifies the transitional urban-rural context facing many primary healthcare systems in Southeast Asia. Nakhon Phanom’s population comprises diverse ethnic groups, including Thai-Lao, Thai-Saek, and Thai-Nyo, each with distinct cultural practices influencing health behaviors and healthcare utilization patterns within primary care settings [12]. In this study, six Thai-Saek communities in At Samat subdistrict, Mueang district, and four Thai-Nyo communities in Phon Sawan subdistrict, Phon Sawan district, were selected as the primary ethnic settings. Two subdistricts were therefore included: Phon Sawan, representing a predominantly rural context with strong traditional social structures served by a community hospital network, and At Samat, a peri-urban area with a mixed agricultural and service-sector economy where residents mainly access care through primary care units linked to the provincial hospital. Across these two subdistricts, a total of 80 participants at risk of type 2 diabetes were recruited, supported by 15 village health volunteers (VHVs) from these ethnic communities who served as trained facilitators.

### Study design and setting

This mixed-methods single-arm pre-post intervention study with convergent design integrated quantitative health outcome measurements with qualitative process evaluation to examine primary healthcare transformation. The study was conducted from 12 May to 4 July 2025 (eight-week period) and followed up at 12th and 24th Weeks (1 August and 24 October 2025) in two sub-districts of Nakhon Phanom Province: Phon Sawan Sub-district (Phon Sawan district) and At Samat Sub-district (Mueang district). These sites were selected through purposive sampling to represent diverse socio-demographic characteristics and primary healthcare infrastructure configurations.

Phon Sawan Sub-district, characterized by predominantly rural settlement patterns and agricultural livelihoods (population approximately 8,500), accesses primary healthcare through a 30-bed community hospital system with two primary care units. At Samat Sub-district, representing peri-urban development with mixed occupational profiles (population approximately 12,000), utilizes three primary care units linked to the 200-bed provincial hospital. This diversity enabled transformation processes across different primary healthcare system configurations.

### Participants and recruitment

**Fig. 1:**
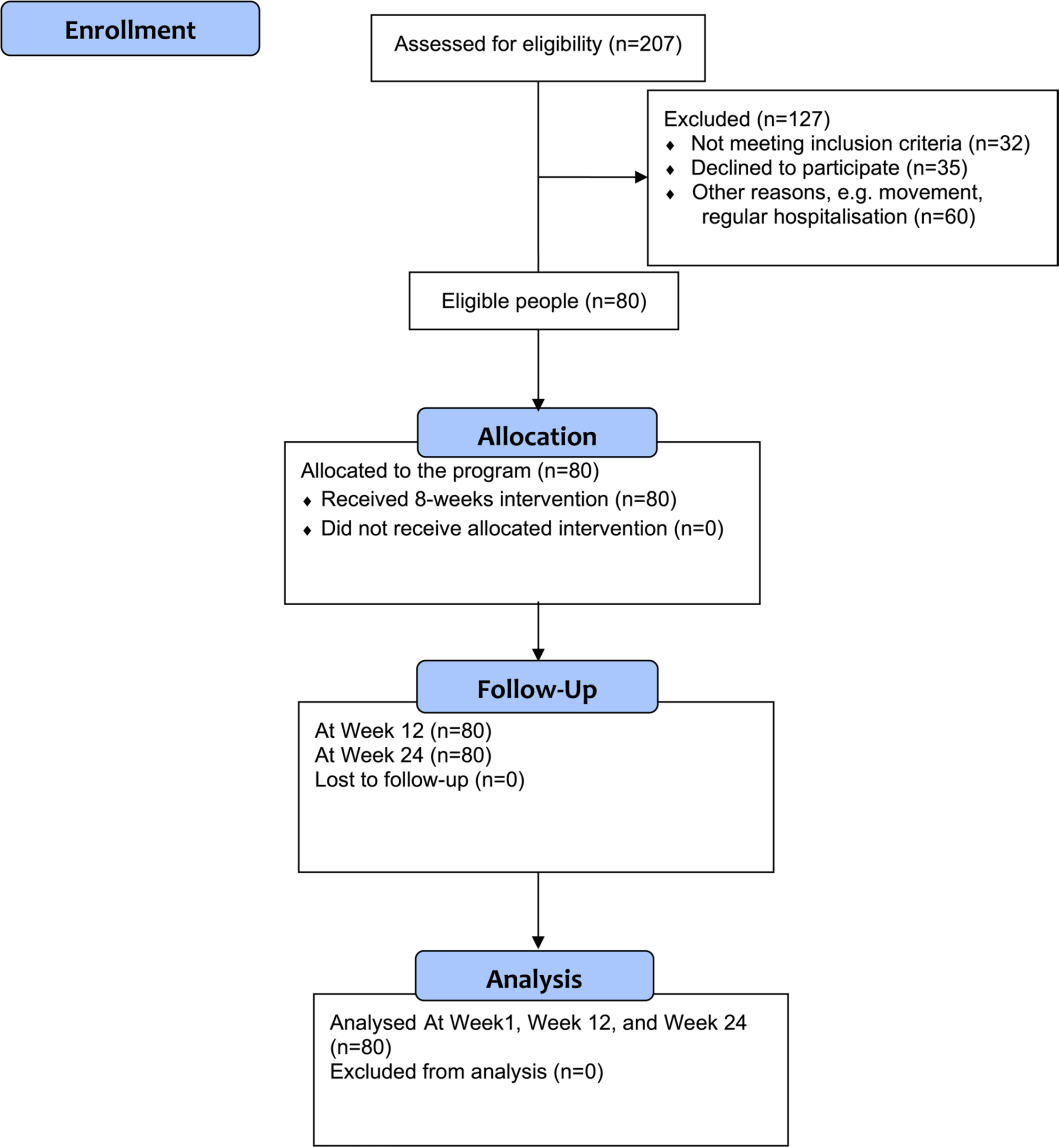
Flow Diagram of the study

There were 80 people recruited from a list of people who were screened for diabetes from the two selected sub-districts. Eligibility criteria included: (1) age 30–70 years, (2) residence in study areas for ≥ 2 years, (3) willingness to participate in 8-week intensive intervention, and (4) no diagnosed diabetes at baseline. Participants were stratified into three spectrum groups based on diabetes risk profiles and intervention needed: Spectrum 1 (*n* = 25): Pre-diabetes with multiple risk factors requiring intensive personalized intervention integrated with primary care (people with a history of Covid-19 infection and with a family history of diabetes); Spectrum 2 (*n* = 25): Moderate risk requiring structured lifestyle modification with primary care monitoring (people with a body mass index above the standard or obese); and Spectrum 3 (*n* = 30): Low-moderate risk benefiting from group-based health literacy and primary care linkage (people who had a dextrose strip blood test with abnormal results but had not yet been diagnosed with diabetes). The recruited people participated in the Community-driven type 2 diabetes prevention through primary healthcare transformation (CDDP-model) for eight weeks and were followed up at 12th and 24th Weeks. See Fig. 1.

### Community-driven type 2 diabetes prevention through primary healthcare transformation: a five-dimensional intervention model (CDDP-model)

The CDDP-model integrated five transformation dimensions during eight-week intensive intervention designed to strengthen WHO SBBs [6] within primary care contexts:

#### Dimension 1: diabetes literacy enhancement in primary care

 This dimension focused on improving community and provider understanding of diabetes prevention through culturally adapted education embedded in primary care services. Activities included community-based workshops (12 sessions, each attended by approximately 45–78 participants), cooking demonstrations using locally available foods (8 sessions), traditional medicine integration consultations for local food menus and dietary guidelines by a Thai traditional doctor (6 sessions), and primary care provider–led health talks in villages and health centers (10 sessions). Educational materials were developed in local languages (Thai-Lao and Thai-Saek) and explicitly incorporated community beliefs about health, diet, and illness causation to enhance cultural resonance. Before initiating community activities, primary care staff completed a 16-hour training program (two full-day sessions) by the researchers (BP, AU and NP) on culturally competent diabetes education and facilitation skills. Dimension 1 activities were delivered throughout the eight-week intervention period in both study sites.

#### Dimension 2: personalized and precision care integrated with primary care

Risk-stratified interventions linked to primary care, including body composition analysis with bioelectrical impedance (InBody 270, Week 1, Week 12, and Week 24), individualized nutrition counseling by primary care nutritionists (three sessions per participant), personalized exercise prescriptions developed with primary care physiotherapists (two consultations), and genetic risk counseling for high-risk participants (*n* = 15 referred from primary care). Primary care electronic health records integrated individual risk profiles and intervention plans.

#### Dimension 3: community–primary health services integration

 This dimension aimed to strengthen linkages between communities and primary care services through coordinated outreach and participatory structures. A mobile health screening camp, organized by primary care staff in Week 2 of the intervention, was used to identify adults at risk of T2D and to invite them to enroll in the program. People at risk of T2D were expected to complete the baseline assessments prior to the commencement of Week 1 activities. In addition, 15 VHVs trained by the researchers were identified to be facilitators for the participants.

The trained VHVs, supervised by primary care staff, conducted home visits throughout the 8-week intervention, with an average of 2.5 visits per participant. These visits focused on reinforcing diabetes prevention messages, monitoring risk factors, and linking participants to primary care services when needed. Peer support groups, facilitated jointly by community leaders and primary care staff, were maintained continuously over the same eight-week period (10 groups in total) and met on a bi-weekly basis to provide mutual support, shared problem-solving, and adherence encouragement.

To embed these activities within local governance structures, community health committees were established in each site within the first four weeks of the intervention (five committees in total). The committees met monthly throughout the eight-week period and served as formal platforms for community–primary care liaison, enabling joint review of local health issues, coordination of activities, and shared decision-making regarding ongoing diabetes prevention efforts.

#### Dimension 4: multi-level health services hub

 Integration of primary, secondary, and tertiary care through: digital health platform connecting primary care units with provincial hospital (secure messaging, referral system), telemedicine consultations for complex cases (*n* = 28 consultations from primary care to specialists), referral protocols between community, primary care, and hospital levels (standardized across five primary care units), and capacity building for primary care providers in diabetes prevention (40-hour training program, *n* = 25 providers).

Digital health platform (https://dm-covid19.prithailand.org/) was designed for high accessibility and real-time community engagement: (1) Communication Layer: A dedicated LINE Messenger Group was established as the primary communication hub. This platform was chosen due to its ubiquitous use as a real-time channel for 15 facilitators to report daily observations, share photos of local ethnic meals (Isan diet), and receive immediate clinical guidance from the research team; and (2) Data management layer: Quantitative data, including fasting blood glucose (FBG) levels and body metrics, were synchronized from the field reports into a centralized digital dashboard (developed via Google Sheets/Looker Studio). This allowed the research team to visualize the progress of participants across the spectrum groups instantaneously. This infrastructure requires no additional hardware beyond standard smartphones and basic internet connectivity, ensuring that the model is highly scalable and feasible for other resource-limited primary care settings.

#### Dimension 5: community and personal monitoring systems 

 Development of integrated monitoring infrastructure including: community health dashboards accessible to participants and primary care (web-based platform), personal health tracking applications linked to primary care records (mobile application, 78% adoption rate), regular monitoring by community health workers reporting to primary care (bi-weekly assessments), and quarterly community health forums presenting data to stakeholders including primary care leadership (*n* = 3 forums, 50–85 participants each). See Table 1.

**Table 1:** Weekly activities according to five dimensions of the model

Week	Activities
1	• Initial health assessment (body composition, vital signs, waist circumference and fasting blood glucose) • Health literacy assessment • Motivation building and behavior change goal setting
2	• Education on new diabetes paradigm (physician instructor) • Training in appropriate dietary control (nutritionist instructor) • Training on health status recording in guidebook • Goal setting and activity selection
3	• Weekly health status assessment • Training in physical activity and appropriate exercise (sports scientist instructor) • Training in adequate rest and self-care activities (mental health team instructor) • Health record verification
4	• Weekly health status assessment • Buddy system pairing for positive health reinforcement • Record verification and feedback
5	• Weekly health status assessment • Training in eating out strategies • Record verification and feedback
6	• Weekly health status assessment • Training in illness preparedness • Record verification and feedback
7	• Weekly health status assessment • Training in finding long-term health motivation • Record verification and feedback
8	• Post-program health assessment (body composition, vital signs, waist circumference, fasting blood glucose) • Review of nutrition, exercise, and rest practices • Record verification and feedback • Positive reinforcement and rewards

### Data collection and measures

Quantitative data were collected at baseline (1st Week), 12th Week and 24th Week through primary care facilities by trained staff using standardized protocols. Anthropometric measurements included height (stadiometer, nearest 0.1 cm), weight (digital scale, nearest 0.1 kg), waist circumference (measuring tape at midpoint between lowest rib iliac crest, nearest 0.1 cm), and body composition (fat mass, muscle mass, visceral fat level) assessed with using bioelectrical impedance analysis. Clinical measures comprised FBG (a glucometer, Accu-Chek Performa (Roche Diagnostics, following an overnight fast of at least eight hours and abstinence from alcohol), blood pressure and resting heart rate (using an electronic device). All electronic devices were calibrated annually by the Nakhon Phanom Provincial Hospital. Body composition and clinical measures were assessed using a single measurement between 6:00 and 8:00 a.m. on the scheduled date. In addition, BMI was calculated as weight(kg)/height(m²).

Quantitative questionnaires covered diabetes prevention knowledge, Physical activity, and Dietary behaviors. Content validity was reviewed by a panel of three diabetes experts, yielding an Item-Objective Congruence (IOC) index of 0.87.

Diabetes prevention knowledge was assessed using a 20-item questionnaire developed by the research team based on key content from the Thai national clinical practice guidelines for diabetes, covering domains such as risk factors, preventive behaviors, screening, and complications. Each correct response was scored as one point (incorrect or “don’t know” = zero), yielding a total score range of 0–20. Higher scores indicated better diabetes prevention knowledge, and scores ≥ 15 were classified as adequate knowledge. The scale demonstrated good internal consistency in this study (Kuder-Richardson Formula 20 = 0.82).​.

Physical activity was measured using a questionnaire developed by the researchers, following standard scoring procedures to calculate total weekly energy expenditure in MET-minutes. Participants were categorized into low (< 600 MET-min/week), moderate (600–2999 MET-min/week), or high (≥ 3000 MET-min/week) physical activity levels according to established IPAQ-SF cut-off points.

Dietary behaviors were evaluated using a Thai food frequency questionnaire (FFQ) adapted for the northeastern region by the researchers, comprising 35 items that assessed the weekly frequency of consumption of staple foods, vegetables, fruits, sugar-sweetened beverages, fried foods, and traditional local dishes. Responses were scored on a 0–4 scale (never to ≥ 6 times per week) to derive a composite dietary behavior score ranging from 0 to 140, with higher scores representing healthier dietary patterns. The FFQ was adapted from tools used in the Thailand National Health Examination Survey and further reviewed by local nutrition experts for cultural and regional appropriateness. The Cronbach’s α coefficient was > 0.70.

### Qualitative data collection

 Employed multiple methods to examine primary healthcare transformation processes: (1) Focus group discussions (FGDs) with participants stratified by spectrum group (10 groups, 6–8 participants each, 60–90 min, audio-recorded and transcribed); (2) Key informant interviews with community health workers (*n* = 15), primary care providers (*n* = 12), community leaders (*n* = 8), and local government officials (*n* = 5); (3) Observation of intervention activities including primary care consultations, community workshops, and health committee meetings (*n* = 35 observations, structured field notes); (4) Document analysis of primary care records, community meeting minutes, and intervention materials. The FGDs, interviews and document analysis were conducted at 25th Week by BP, AU, KC, PK and NP, while the Observation was done during the 1st Week and 24th Weeks by BP, WB, and PW. BP, AU, WB, PK were female and PK, KC, PW and NP were male. Key informants for FGDs and interviews were purposively selected and selection was stopped after the interviewers agreed that there was no more information came from the last key informant. The guides for focus group discussions, interviews, observations, and records were constructed by the researchers (Supplementary file).

In addition, budget and spent money related to the CDDP model was collected to assess the unit cost for conducting the CDDP model.

### Data analysis

Descriptive statistics characterized participant demographics and baseline health status. Paired t-tests assessed changes in continuous outcomes from baseline (1st Week) to 12th and 24th Weeks. Analysis of variance (ANOVA) compared outcomes across spectrum groups, while repeated ANOVA and post hoc comparison compared study outcomes across times (1st Week, 12th Week, and 24th Week). The study outcomes were clinical and behavioral outcomes (waist circumference, FBG, body fat percentage, physical activity levels, and diabetes prevention knowledge). Assumptions for paired t-tests, ANOVA, and repeated ANOVA (e.g. normality, independence of observations) were checked before doing inferential analysis. Tukey’s Honestly Significant Difference was used for post hoc analysis. Effect sizes (Cohen’s d) quantified magnitude of changes. All statistical tests using SPSS version 28.0 were two-tailed, and a significance level of alpha = 0.05 and 95% confidence intervals (CI) were used to determine and report statistical significance. Missing data (< 5% for all variables) were handled using multiple imputations.

Thematic analysis following Braun and Clarke’s six-phase approach was employed [13]. Two researchers (AU and PK) independently coded transcripts using NVivo 14 software. We (BP, PK and NP) employed a hybrid deductive and inductive approach. Initially, the WHO SBBs framework was used deductively to provide a structural guide for the initial coding process. Thereafter, we allowed inductive coding to capture emergent themes that did not fit strictly within the pre-defined SBB categories. This ensured that the unique cultural and community-specific nuances of the CDDP model were preserved and not forced into a rigid framework. Themes were reviewed and refined through team discussion. Final themes were defined and exemplary quotes selected. Trustworthiness was enhanced through: investigator triangulation (multiple coders), data triangulation (multiple sources), member checking with participants (*n* = 20 reviewed transcripts), and maintenance of audit trail. Analysis specifically examined transformation processes in primary care governance, information systems, resource mobilization, service delivery networks, health workforce, and medical supplies/technology.

#### Integration of quantitative and qualitative data

 A mixed-methods single-arm pre-post intervention study with convergent design allowed independent collection and analysis followed by integration. Integration occurred through: (1) data collection both quantitative data (e.g. from the questionnaire) and qualitative data (e.g. FGDs), (2) analysis linking between quantitative outcomes with qualitative themes for each WHO building block in primary care, and (3) narrative synthesis explaining how transformation processes led to observed health outcomes within primary care systems.

## Results

### Participant characteristics

There were 80 participants who completed the eight-week intervention (100% retention). Mean age was 54.3 years (standard deviation: SD ± 8.6), with 97.5% female. Educational attainment included primary school (35%), secondary school (42%), and tertiary education (23%). Occupational distribution comprised agriculture (38%), government service (22%), small business (18%), and informal sector (22%). Baseline health status showed: mean Body Mass Index (BMI) 32.5 kg/m² (SD ± 5.4), mean waist circumference 85.5 cm (SD ± 10.0), mean FBG 107.4 mg/dL (SD ± 23.5), with 8.6% classified as pre-diabetic. Prior primary care utilization averaged 2.3 visits/year (SD ± 1.8). See Table 2.

**Table 2:** Baseline characteristics of the participants

Characteristic	*n* = 80
Age, years, mean (SD)	54.3 (8.6) ​
Female, n (%)	78 (97.5) ​
Body Mass Index, kg/m², mean (SD)	32.5 (5.4) ​
Waist circumference, cm, mean (SD)	85.5 (10.0) ​
Fasting blood glucose, mg/dL, mean (SD)	107.4 (23.5) ​
Pre-diabetes (100–125 mg/dL), n (%)	25 (31.2) ​
High fat mass	47 (58.7)
Low muscle mass	53 (66.2)

To assess the feasibility of scaling the CDDP model, a cost analysis was conducted. The total intervention cost was approximately 30,000 THB (about 850 US dollars) for 80 participants over eight weeks. The costs included: Competency training for 15 facilitators, Ongoing operational costs, glucose monitoring supplies and the maintenance of the digital dashboard. This equated to a cost/person of 375 THB (about 11 US dollars).

### Clinical and behavioral outcomes in primary care

**Fig. 2:**
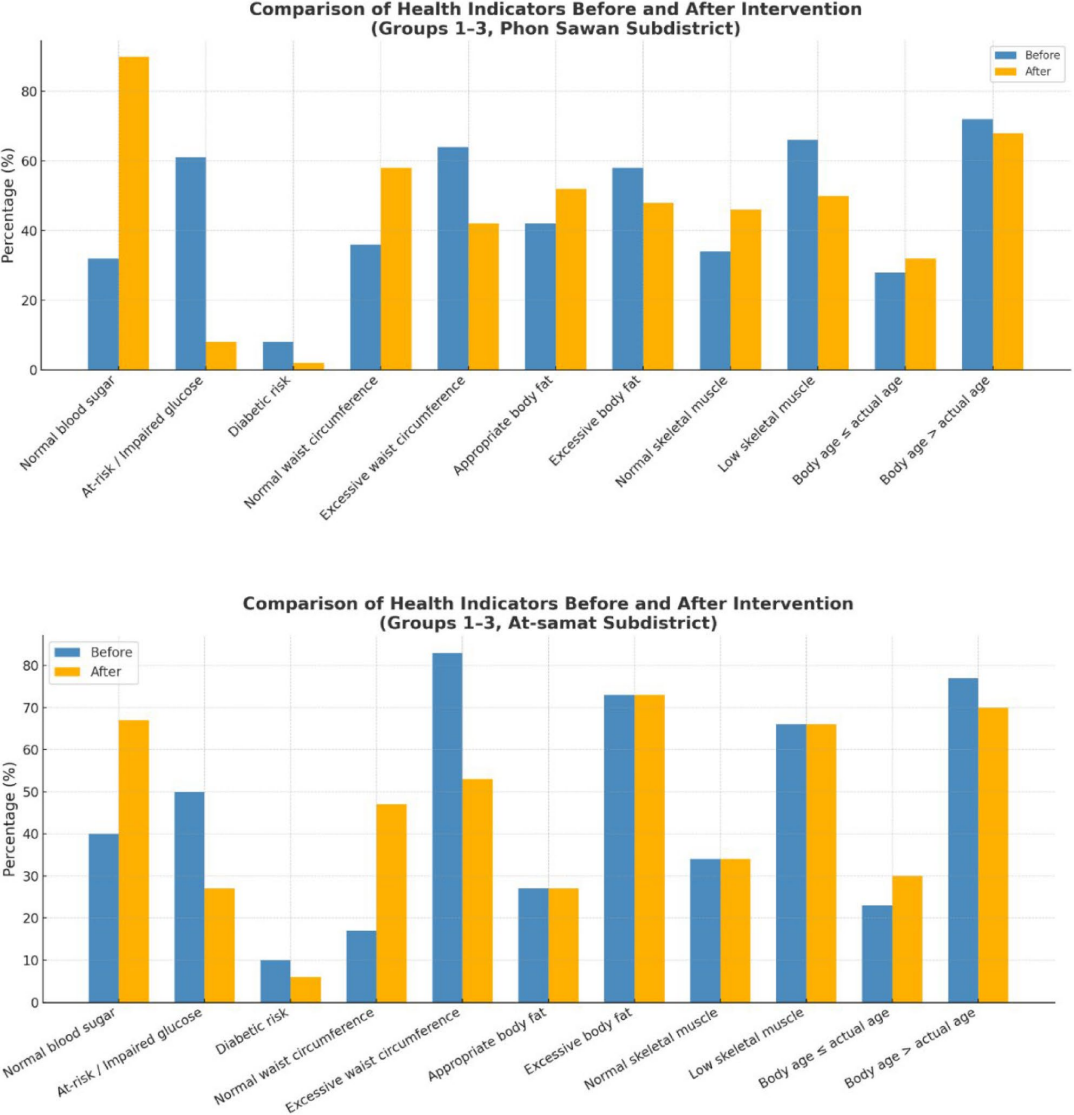
Comparisons of health indicators before and after intervention among two sub-districts

Significant improvements were observed across multiple health indicators monitored through primary care. Mean waist circumference decreased by 8.2 cm (95% CI: 7.1–9.3, *p* < .001, Cohen’s d = 1.24). Mean FBG decreased by 15.3 mg/dL (95% CI: 12.8–17.8, *p* < .001, Cohen’s d = 1.47) among pre-diabetic participants. Mean body fat percentage decreased by 3.8% (95% CI: 3.1–4.5, *p* < .001, Cohen’s d = 0.89). Mean physical activity levels increased by 850 MET-minutes/week (95% CI: 680–1020, *p* < .001, Cohen’s d = 1.05). Diabetes prevention knowledge scores increased from 10.25 to 14.93 (Mean difference = 4.68) or increased by 23.4% (95% CI: 20.1–26.7, *p* < .001, Cohen’s d = 1.88). Spectrum-specific from ANOVA analysis revealed differential outcomes. Spectrum 1 (intensive intervention) showed the largest improvements in FBG (-21.4 mg/dL, *p* < .001) and waist circumference (-11.3 cm, *p* < .001). Spectrums 2–3 showed some improvements across indicators. See Fig. 2.

From the post-hoc analysis, the body weight, BMI, fat mass, basal metabolic rate (BMR) reduced significantly from baseline to 12th Week (Mean Difference = 1.946, *p*<.001, Mean Difference = 1.162, *p*<.001, Mean Difference = 0.912, *p* = .028, Mean Difference = 19.59, *p*<.001, respectively) and remained the same from 12th Week to 24th Week (*p*=.349, *p*=.617, *p*=.445, *p*=.067, respectively). In addition, FBG showed no significant difference between baseline and 12th Week (*p*=.144) but showed significant difference between baseline and 24th Week (*p*=.028). Amongst waist circumference, visceral fat level and muscle mass, there was no significant difference from baseline to 24th Week (F(2,158) = 1.27, *p*=.285, F(2,158) = 1.04, *p*=.355, and F(2,158) = 0.812, *p*=.446, respectively). See Table 3.

**Table 3:** Comparisons of health indicators between times

Health indicators	F	df	*p*-value	Effect size	Post-hoc comparison		
					Baseline and 12th Week	Baseline and 24th Week	12th Week and 24th Week
Body weight	24.3	2,158	< 0.001	0.235	Significant difference	Significant difference	No significant difference
Body mass index	15.1	2,158	< 0.001	0.16	Significant difference	Significant difference	No significant difference
Waist circumference	1.27	2,158	0.285	0.016	No significant difference	No significant difference	Significant difference
Fat mass	4.8	2,158	0.009	0.057	Significant difference	No significant difference	No significant difference
Visceral fat level	1.04	2,158	0.355	0.013	No significant difference	No significant difference	No significant difference
Muscle mass	0.812	2,158	0.446	0.01	No significant difference	No significant difference	No significant difference
Basal metabolic rate	10.8	2,158	< 0.001	0.121	Significant difference	Significant difference	No significant difference
Fasting blood glucose	4.95	2,100	0.009	0.09	No significant difference	Significant difference	No significant difference

*df* degree of freedom

### Primary healthcare system transformation: WHO building blocks analysis

Qualitative analysis revealed transformation across all WHO SBBs within primary care contexts:

#### Building Block 1: leadership and governance in primary care

 Governance structures changed from providers’ decision-making to participatory co-design. Community health committees established formal mechanisms for community input to primary care planning, with 85% of participants reporting increased engagement (from review of each individual record).

One community leader stated: “Previously, primary care has decided everything for us. Now we sit together, discuss priorities, and make decisions jointly”.

Primary care providers described mindset shifts: “We learned to listen first rather than prescribe. Communities know their needs better than we assumed”. New governance mechanisms included community representation in primary care unit management committees (*n* = 5 units), quarterly participatory planning workshops, and community-approved annual workplans.

#### Building Block 2: health information systems 

 Information systems evolved from fragmented individual records to more integrated community-wide monitoring platforms accessible to primary care. Digital dashboards provided real-time visualization of community health indicators (mainly individual outcomes), enabling primary care providers to identify trends and target of interventions (https://dm-covid19.prithailand.org/).

One primary care physician noted: “The dashboard changed how we work. Instead of waiting for patients to come, we can see which communities need proactive outreach”.

Community members accessed personal health data through mobile applications linked to primary care records, with 78% reporting the technology helped track progress. Primary care electronic health records were enhanced to include risk stratification, intervention tracking, and community-level analytics.

#### Building Block 3: resource mobilization and financing 

Local resource management expanded through multi-sectoral partnerships coordinated by primary care. Two municipalities allocated additional budget for community diabetes prevention (total 450,000 baht; about 14000 US dollars). Eight local businesses provided in-kind support including healthy food donations and venue space. Five civil society organizations contributed volunteer time and expertise.

One primary care provider explained: “We learned resources exist in our communities if we know how to manage them. It’s not just about government budgets”.

Estimated value of managed resources reached 1.2 million baht beyond programmatic budget, demonstrating a more sustained initiative for primary care.

#### Building Block 4: service delivery networks

 Service delivery networks successfully integrated primary, secondary, and tertiary care levels through digital connectivity and formalized referral protocols. Telemedicine consultations enabled primary care providers to access specialist physicians for complex cases (*n* = 28 consultations, 95% resolved without physical referral). Standardized referral protocols across five primary care units improved care coordination by meetings and agreements of specialist physicians, nutritionists, registered nurses, physiotherapists, village leaders and primary care providers.

One participant described: “When my blood sugar was concerning, the VHV coordinated with primary care providers, they consulted with hospital doctor through video, and I received comprehensive care without leaving the community”.

Bi-directional communication between VHVs and primary care providers strengthened through structured reporting systems and regular case conferences.

#### Building Block 5: health workforce transformation 

Significant health workforce transformation occurred across multiple levels within and beyond primary care. VHVs (*n* = 15) were upskilled as health literacy facilitators through 40-hour training covering diabetes pathophysiology, risk assessment, behavior change counseling, and community mobilization. Primary care staff (*n* = 25) received training in culturally competent diabetes prevention, participatory planning, and community engagement.

One VHV stated: “The training transformed my identity from volunteer to professional health educator working alongside primary care”.

Traditional healers (*n* = 6) were integrated into primary care teams through collaborative consultations. Role expansion enabled primary care to extend reach through trained community workforce, addressing provider shortages in rural areas.

#### Building Block 6: medical products, vaccines, and technologies 

 Technology adoption encompassed multiple innovations integrated into primary care: body composition analyzers (*n* = 5 units distributed to primary care facilities), digital health platform connecting stakeholders (one web-based platform and one mobile application), telemedicine equipment enabling remote consultations from primary care (videoconferencing systems in five primary care units), and point-of-care testing devices for glucose and HbA1c (*n* = 5 units).

One primary care provider noted: “Technology bridged the gap between community and hospital. We can provide sophisticated assessments at primary care level now”.

Challenges included initial learning curves and internet connectivity issues in rural areas, addressed through intensive training and mobile data support.

### Mechanisms of sustained change in primary care

Qualitative analysis identified three key mechanisms enabling sustained transformation within primary healthcare systems:

#### Community ownership and empowerment

Participants described shifts from passive service recipients to active co-producers of health alongside primary care. One participant stated: "We are not waiting for primary care to solve our problems. We are solving them together". Community ownership manifested through voluntary contributions, peer support, and collective problem-solving. Primary care providers recognized communities as partners: "We provide expertise, but communities provide context, relationships, and sustainability that we cannot".

#### Peer support networks integrated with primary care

Peer support groups (n=10) created social engagement and mutual encouragement linked to primary care follow-up.Participants emphasized peer influence: "When friends in my group succeed, it motivates me. We encourage each other between primary care visits".Groups continued meetings beyond intervention period, peer support has continued within primary care systems.

#### Multi-sectoral partnerships

Partnerships between primary care, local government, private sector, and civil society created synergistic effects. Coordinated efforts addressed social determinants beyond primary care's capacity alone.One municipal official noted: "Diabetes prevention is not just a health sector issue. It requires coordinated action across sectors, with primary care as the hub".

## Discussion

### Principal findings: Primary healthcare transformation through community engagement

This study demonstrates that community-driven diabetes prevention programs integrated within primary healthcare can support meaningful improvements across WHO SBBs when designed with intentional community engagement processes. The five-dimensional intervention model successfully generated improvements across clinical outcomes, behavioral changes, and system-level transformations within primary care contexts. Also, the study illuminates pathways for repositioning communities from passive service recipients to active co-producers of health alongside primary care providers—representing the essence of primary healthcare transformation [14, 15].

The integration of transformation change theory with WHO SBBs framework provided a practical analytical lens for understanding how disease-specific interventions in primary care can leverage and reinforce system-wide improvements. Unlike conventional diabetes prevention programs that remain siloed within clinical services, this approach embedded prevention within broader primary healthcare strengthening agendas, addressing governance, information systems, resource mobilization, service networks, workforce development, and technology adoption simultaneously [16].

### Comparison with existing literature on primary care transformation

Our findings align with growing evidence that community engagement strengthens primary healthcare systems beyond disease-specific outcomes [17, 18]. However, this study extends existing literature by systematically analyzing transformation across all WHO SBBs within primary care contexts. Previous community-based diabetes prevention studies in primary care settings have demonstrated clinical efficacy [19, 20] but rarely examined health system transformation mechanisms. Our mixed-methods approach revealed how community engagement catalyzes systemic changes in governance, information systems, and resource mobilization—dimensions typically overlooked in clinical trials but essential for sustainable primary care strengthening [21].

The role of community health workers as bridges between primary care and communities emerged as particularly critical. Systematic investment in this cadre offers high returns for primary healthcare system strengthening [22, 23]. Our study contributes evidence on specific mechanisms through which community health workers facilitate transformation: translating between professional and experiential knowledge systems, mobilizing community resources complementing formal primary care services, and enabling participatory governance connecting communities to primary care decision-making.

Technology adoption within primary care contexts presents both opportunities and challenges. While digital health platforms and telemedicine enhanced primary care connectivity to specialists and enabled real-time community health monitoring, implementation challenges included learning curves, connectivity issues, and digital literacy gaps [24]. Successful technology integration required intensive training, ongoing technical support, and careful attention to user experience within primary care workflows.

### Implications for primary healthcare practice and policy

This study offers several implications for primary healthcare practice and policy. First, diabetes prevention programming should be reconceptualized as opportunities for comprehensive primary healthcare system strengthening rather than standalone clinical interventions. Policymakers should incentivize integrated approaches that simultaneously address multiple WHO building blocks within primary care rather than funding vertical disease-specific programs [25].

Second, transformation requires intentional redistribution of power and resources toward communities and primary care providers. This includes establishing formal mechanisms for community participation in primary care governance, investing in VHVs for specific training and support, and devolving decision-making authority to local primary care levels where appropriate. Top-down planning processes, even when well-intentioned, undermine the community ownership essential for sustained primary care transformation [26].

Third, technology adoption in primary care should prioritize user-centered design and address connectivity barriers. Digital health innovations must complement rather than replace community relationships and primary care provider judgment. Technology should empower communities and primary care providers rather than creating new dependencies or widening health inequities [27].

Fourth, multi-sectoral collaboration is essential for addressing social determinants of health. Formal partnerships connecting primary care with local government, private sector, and civil society can mobilize resources and coordinate actions that individual sectors cannot achieve alone [28]. Primary care leaders should actively cultivate these partnerships as core functions rather than optional activities.

### Strengths and limitations

Study strengths include the mixed-methods design providing comprehensive understanding of both outcomes and transformation processes within primary care; the theoretical grounding in transformation change theory and WHO building blocks framework enabling systematic analysis; the diverse primary care settings (rural and peri-urban) enhancing transferability; and the high retention rate (100%) demonstrating intervention acceptability in primary care contexts.

Several limitations warrant acknowledgment. The eight-week intervention period demonstrated initial transformation processes within primary care but limits conclusions about long-term sustainability. Extended follow-up is necessary to assess whether observed changes persist after external support diminishes. The study’s focus on two sub-districts enhances contextual depth but constrains generalizability to primary care settings with different socio-cultural characteristics or health system configurations. Selection bias may have influenced outcomes, as participants self-selected into the program and likely possessed higher motivation than general populations accessing primary care. Furthermore, the marked gender imbalance in the sample (97.5% female participants) limits generalizability to male populations and should be discussed in future research. The absence of control communities precludes definitive attribution of outcomes to intervention components versus secular trends in primary care development. Resource intensity of personalized interventions and digital infrastructure may challenge scalability in resource-constrained primary care settings without proportional external support. Measurement limitations include reliance on self-reported behavioral data subject to social desirability bias and absence of long-term clinical outcomes (e.g., diabetes incidence rates). Qualitative findings, while providing rich contextual understanding of primary care transformation, may not capture perspectives of non-participants or those who withdrew from engagement with primary care services.

### Future research directions in primary healthcare

Future research should examine long-term sustainability through multi-year follow-up assessing maintenance of transformation processes and health outcomes within primary care systems. Cost-effectiveness analysis comparing transformation approaches versus conventional diabetes prevention programs in primary care needs more details of investigation. It would inform resource allocation decisions. Scalability investigations should examine how intervention intensity, digital infrastructure requirements, and community capacity-building can be adapted for different resource contexts in primary care. Comparative effectiveness research exploring which intervention dimensions most powerfully drive primary care transformation across different cultural and health system contexts would enable evidence-based adaptation. Implementation science studies identifying mechanisms through which transformation change processes can be intentionally catalyzed and supported by primary healthcare leadership would guide scale-up efforts.

## Conclusion

This pre-post study suggests that the community-driven type 2 diabetes prevention program is feasible and may improve health outcomes for people at high risk of T2D in primary healthcare settings in short-term improvements in selected health outcomes. The five-dimensional intervention model has been integrated into structural changes (governance redistribution, resource reconfiguration), relational shifts (partnership development, multi-sectoral collaboration), and cognitive transformations (knowledge integration, capability development) within primary care contexts. However, due to the absence of a control group and short follow-up, further controlled and long-term studies are needed to confirm effectiveness and system-level impact.

## Supplementary Information

Supplementary Material 1.

## Data Availability

The datasets generated and analyzed during this study are available from the corresponding author upon reasonable request.
