# Supplementary material for: Community-driven type 2 diabetes prevention in primary healthcare: a mixed-methods pre-post intervention study in Thailand
Source: BMC Prim Care. 2026 May 12;27:250. doi: 10.1186/s12875-026-03362-x (PMC13326121; doi:10.1186/s12875-026-03362-x)
Supplement: Supplementary file 1 — Supplementary Material 1. [file 12875_2026_3362_MOESM1_ESM.pdf]

Set No. .... (for Researchers)

Diabetes Risk Screening Tool

Section 1: Personal History and Basic Physical Examination

1.1 Sex at birth: ☐ Male ☐ Female

1.2 Age: .... years

1.3 Height: .... cm

Weight: .... kg

1.4 Waist circumference: .... cm

1.5 Blood pressure: Systolic .... / Diastolic .... mmHg

1.6 Chronic diseases: ☐ None ☐ Yes, specify: ....

1.7 Does any family member have diabetes?

☐ No ☐ Yes

☐ Grandfather ☐ Grandmother ☐ Paternal grandfather ☐ Paternal grandmother

☐ Father ☐ Mother ☐ Sibling ☐ Sibling

1.8 Have you ever been infected with COVID-19? ☐ Yes ☐ No

1.9 History of health conditions at risk for diabetes:

Polycystic ovary syndrome: ☐ Yes ☐ No

Hyperlipidemia: ☐ Yes ☐ No

History of gestational diabetes or delivery of infant weighing >4 kg: ☐ Yes ☐ No

Section 2: Smoking and Alcohol Consumption Behaviors

2.1 Do you currently smoke? ☐ Yes ☐ No

2.2 Do you currently consume alcohol (liquor/beer/alcoholic beverages)? ☐ Yes ☐ No

Section 3: Food and Beverage Consumption Behaviors

3.1 Do you consume salty foods? ☐ Salty ☐ Not salty

3.2 Do you consume sweet foods? ☐ Sweet ☐ Not sweet

3.3 Do you add condiments to food? ☐ Yes ☐ No

(fish sauce, sugar, chili, vinegar, chili fish sauce)

3.4 Do you add sugar and non-dairy creamer to coffee? (including 3-in-1 coffee) ☐ Yes ☐ No

3.5 Do you drink soft drinks? ☐ Frequently ☐ Infrequently

3.6 Do you consume more than 3 spoonfuls/3 fistfuls (of your own hand) of vegetables per day?

☐ More than ☐ Less than or equal to

3.7 Do you consume more than 2 spoonfuls/2 fistfuls (of your own hand) of fruit per day?

☐ More than ☐ Less than or equal to

Section 4: Physical Activity and Rest Behaviors

4.1 Do you exercise regularly? (at least 45 minutes total per week) ☐ Yes ☐ No

4.2 Do you get sufficient sleep? (at least 7 hours per day) ☐ More than ☐ Less than

Interview Guide Questions (for At-Risk Diabetes Groups)

1. What is your knowledge and understanding of energy intake from food consumption?
2. Do you think carbohydrates or starchy foods affect your blood sugar levels? If so, how?
3. What do you think is the impact of physical exercise on the body?
4. What form or method do you think would be most appropriate for building understanding of self-practices to prevent diabetes for yourself and your neighbors?
5. What are your opinions on self-care regarding diet specification, physical exercise, and sufficient rest?
6. How do you think you can participate in planning to enhance health literacy for yourself and neighbors in the community to avoid or stay safe from diabetes?

Focus Group Discussion Guide (for At-Risk Diabetes Groups)

1. What is your knowledge and understanding of diabetes, including its benefits or harms?
2. How do you define at-risk groups for diabetes? What are the causes and prevention strategies to avoid developing diabetes?
3. What form or method do you think would be most appropriate for building understanding of self-practices to prevent diabetes for yourself and your neighbors?
4. What are your opinions on self-care regarding diet specification, physical exercise, and sufficient rest?
5. How do you think you can participate in planning to enhance health literacy for yourself and neighbors in the community to avoid or stay safe from diabetes?
6. From which sources have you searched for or received information on diabetes risk and prevention? What was the content like?
7. Which source or program provided information and practices on diabetes risk and prevention that satisfied you the most, and why?

8. From which medium or source did you remember the details of diabetes prevention practices the most, and why were you able to recall content from that medium/source?
9. How do you discuss information on practices related to diabetes risk and prevention? Which topics were discussed most? Which topics were not put into practice, and why?
10. If accurate information on practices related to diabetes risk and prevention is disseminated to you, what type of media, channel, time period, and methods should be used so you can follow health information continuously and consistently?

Focus Group Discussion Guide (for Subdistrict Health Promoting Hospital Working Team, Possible Stakeholders)

1. What do you think the recruitment format for target at-risk diabetes group members should be to ensure they understand the meaning, rationale, and become aware and confident in self-care to prevent diabetes?
2. What strategies do you think can raise awareness and encourage project participants to seek information, ask questions, and explore options for health care to prevent diabetes, making it feasible in the community setting?
3. What approaches and methods do you think should be used to communicate information to neighbors and volunteer teams?
4. How do you think services to prevent diabetes in your community should be designed?
5. How do you think learning activities at the community level can be continuously organized to align with your community's lifestyle?
6. How should the sequence of task difficulty be prioritized when working with the community to reach at-risk diabetes groups equitably and comprehensively?

Set No. .... (for Researchers)

Health Management Record for Diabetes Prevention

Week No. ……. Month ……………………………………………….. B.E. ………………

Goals (Overall)

1...

2...

3...

Section 1: Food Intake Record

Date .... Month .... B.E. ....

Your Dietary Control Goals

1...

2...

3...

Daily Food Intake Record

Day	Food Item	Amount Consumed	Breakfast	Lunch	Dinner
**Monday**					
**Tuesday**					
**Wednesday**					
**Thursday**					

Day	Food Item	Amount Consumed	Breakfast	Lunch	Dinner
**Friday**					
**Saturday**					
**Sunday**					

Section 2: Physical Activity Record

Date .... Month .... B.E. ....

Your Physical Activity Goals

1. ....
2. ....
3. ....

Exercise Plan to Promote Diabetes Prevention

Day	Training Program (Specify Activity Details)	Week
Monday		
Tuesday		
Wednesday		
Thursday		
Friday		

Set No. .... (for Researchers)

**Day**	**Training Program (Specify Activity Details)**	**Week**
Saturday		

Section 3: Recreation and Rest Record

Date .... Month .... B.E. ....

Your Mental Health Care Goals

1..

2..

3..

Mental Health Care Plan to Promote Diabetes Control and Prevention

**Activity/Day**	**Monday**	**Tuesday**	**Wednesday**	**Thursday**	**Friday**	**Saturday**	**Sunday**
Bedtime – Wake Time							
Sleep Quality<br>☐ 1. Takes long to fall asleep<br>☐ 2. Wakes up and hard to fall back asleep<br>☐ 3. Sound sleep<br>☐ 4. Interrupted sleep	☐1<br>☐2<br>☐3<br>☐4	☐1<br>☐2<br>☐3<br>☐4	☐1 ☐2<br>☐3 ☐4	☐1 ☐2<br>☐3 ☐4	☐1<br>☐2<br>☐3<br>☐4	☐1 ☐2<br>☐3 ☐4	☐1<br>☐2<br>☐3<br>☐4
Recreational Activities (Activities that make you feel relaxed, happy, or smiling)							
Overall Daily Feeling<br>☐ 0 Exhausted, no energy<br>☐ 1 Wilted, unmotivated	☐0<br>☐1<br>☐2<br>☐3	☐0<br>☐1<br>☐2<br>☐3	☐0 ☐1<br>☐2 ☐3<br>☐4 ☐5	☐0 ☐1<br>☐2 ☐3<br>☐4 ☐5	☐0<br>☐1<br>☐2<br>☐3	☐0 ☐1<br>☐2 ☐3<br>☐4 ☐5	☐0<br>☐1<br>☐2<br>☐3

Set No. .... (for Researchers)

Activity/Day	Monday	Tuesday	Wednesday	Thursday	Friday	Saturday	Sunday
☐ 2 Anxious, restless<br>☐ 3 Calm, at ease<br>☐ 4 Refreshed, happy<br>☐ 5 Joyful, fulfilled, energetic	☐4<br>☐5	☐4<br>☐5			☐4<br>☐5		☐4<br>☐5

Body Composition and Analysis Record

Item	Measurement 1 .../..../....		Measurement 2 .../..../....	
	Result	Interpretation	Result	Interpretation
Weight (kg)				
BMI (kg/m²)				
Waist Circumference (cm)				
Body Fat (%)				
Subcutaneous Fat				
Skeletal Muscle				
Body Age				
Visceral Fat Level				
Resting Metabolic Rate (RM)				
Blood Glucose Level (DTX)				
